# Nonunion of a medial malleolar stress fracture in an adolescent athlete secondary to lateral ankle instability: A case report

**DOI:** 10.1016/j.ijscr.2020.12.043

**Published:** 2020-12-25

**Authors:** Kensei Yoshimoto, Masahiko Noguchi, Hideyuki Maruki, Mina Ishibashi, Ken Okazaki

**Affiliations:** a Department of Orthopedic Surgery, Tokyo Women's Medical University, 8-1 Kawadacho, Shinjuku-ku, Tokyo 162-0054, Japan; b Orthopaedic Foot and Ankle Center, Shiseikai Daini Hospital, 5-19-1 Kamisoshigaya, Setagaya-ku, Tokyo, 157-8550, Japan; c Orthopedic Foot and Ankle Center, Daisan, Kitashinagawa Hospital, 3-3-7 Kitashinagawa, Shinagawa-ku, Tokyo 140-0001, Japan

**Keywords:** CLAI, chronic lateral ankle instability, CT, computed tomography, MRI, magnetic resonance imaging, Medial malleolar stress fracture, Medial malleolus, Arthroscopic Broström, Nonunion, Internal fixation, Case report

## Abstract

**Introduction:**

Medial malleolar stress fractures are relatively uncommon. This report describes the successful treatment of nonunion of a medial malleolar stress fracture due to chronic lateral ankle instability.

**Presentation of case:**

A 13-year-old middle school student who belonged to a football club presented to our clinic with chronic medial left ankle pain lasting over a year. He had sprained his left ankle several times 6 years earlier. A plain anteroposterior ankle radiograph showed a vertical fracture line in the medial malleolus involving the epiphyseal plate, and computed tomography demonstrated the vertical fracture seen on the plain radiographs and bone sclerosis at the fracture site. We performed internal fixation for nonunion of the medial malleolar stress fracture with arthroscopic modified Broström for lateral ankle instability. Two years after surgery, the Self-Administered Foot Evaluation Questionnaire improved in all parameters, and both the anterior drawer and varus stress tests were negative.

**Discussion:**

Early diagnosis of medial malleolar stress fracture is important for a rapid return to sports. Magnetic resonance imaging is helpful for early diagnosis. Because lateral ankle instability can cause medial malleolar stress fracture, arthroscopic modified Broström procedure is meaningful for medial malleolar stress fracture with lateral ankle instability.

**Conclusion:**

Internal fixation and the arthroscopic modified Broström procedure could achieve good clinical outcomes for medial malleolar stress fractures with lateral ankle instability.

## Highlights

• A 13-year-old male football player presented with chronic medial left ankle pain.
• Radiographic examination showed nonunion of the medial malleolar stress fracture and chronic lateral ankle instability.
• Internal fixation with the arthroscopic modified Broström procedure was performed.
• The Self-Administered Foot Evaluation Questionnaire improved in all parameters.
• The method is useful for medial malleolar stress fractures due to chronic lateral ankle instability.

## 1 Introduction

Stress fractures are common overuse injuries seen in athletes. They mostly occur in the lower extremities owing to the repetitive weightbearing forces imparted on the bony anatomy, and specific anatomic sites have been associated with individual sports [[1], [2], [3]].

Medial malleolar stress fractures are relatively uncommon, accounting for 0.6%–4.1% of all stress fractures [1,4]. The diagnosis of medial malleolar stress fractures may be difficult because of the vague symptoms and insidious onset. It is important to diagnose this condition early because failure to assess and manage the fracture properly can result in complications such as progression to a complete fracture, delayed union, or nonunion, which lead to chronic pain, and delayed return to sports [5,6].

Of the few reported cases, only one included nonunion of a medial malleolar stress fracture [7]. We present the case of a nonunion of medial malleolar stress fracture in an adolescent football player secondary to chronic lateral ankle instability (CLAI). After successful operative management, including open reduction and internal fixation with an arthroscopic modified Broström procedure [8] to treat the CLAI, he experienced a rapid return to football.

This case is reported in line with the SCARE criteria [9].

## 2 Presentation of case

**Fig. 1:**
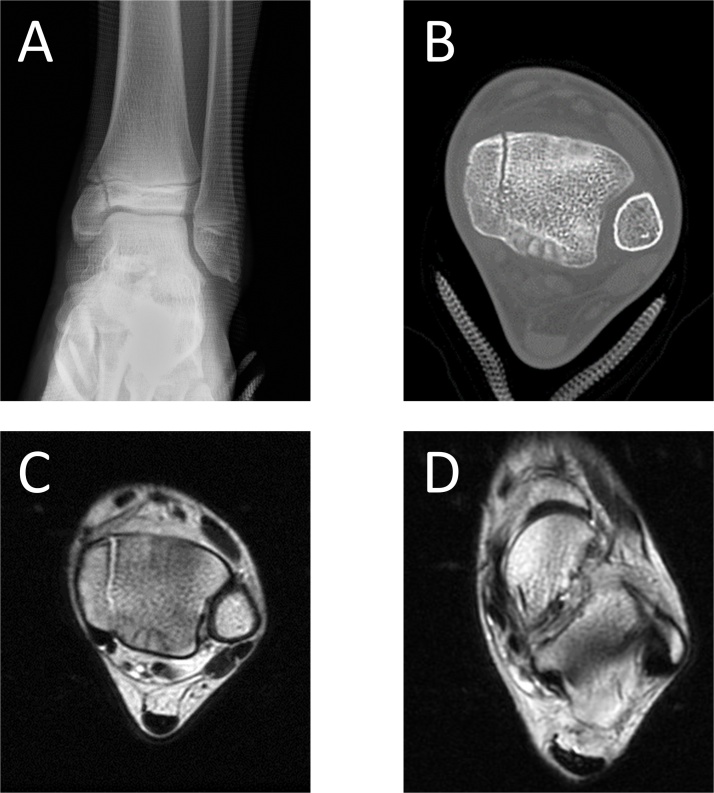
A preoperative plain anteroposterior radiograph of the left ankle showing a vertical fracture line in the medial malleolus (A). Computed tomography showing a bone sclerosis at the fracture site (B). T2-weighted magnetic resonance imaging showing linear high signal intensity in the medial malleolus (C) and the thin anterior talofibular ligament (D).

A Japanese 13-year-old middle school boy (height 167 cm, weight 50 kg) who belonged to a football club presented to our clinic with chronic medial left ankle pain lasting over a year. He had sprained his left ankle several times 6 years earlier. One year before presenting to our clinic, he sprained his ankle again and experienced medial ankle pain with swelling. He visited a neighboring clinic and was diagnosed with ankle sprain. Thereafter, he resumed football. The symptoms were mild at rest, but increased upon walking and training. Three weeks before presenting to our hospital, his ankle pain worsened and he visited another clinic. He was diagnosed with medial malleolar stress fracture, and was referred to our clinic, which is in a secondary emergency hospital. A plain anteroposterior ankle radiograph showed a vertical fracture line in the medial malleolus involving the epiphyseal plate (Fig. 1A).

Advanced imaging studies of the ankle were ordered to assess for additional underlying pathologic features because his symptoms had persisted for over a year. Computed tomography (CT) revealed the fracture seen on the plain radiographs and bone sclerosis at the fracture site (Fig. 1B). T2-weighted magnetic resonance imaging (MRI) showed linear high signal intensity in the medial malleolus and the thin anterior talofibular ligament (Fig. 1C and D).

We considered that the patient’s medial malleolar stress fracture occurred more than 1 year previously, secondary to CLAI, and was not fused. Therefore, we planned open reduction and internal fixation of the medial malleolus using an arthroscopic modified Broström procedure to correct the CLAI.

**Fig. 2:**
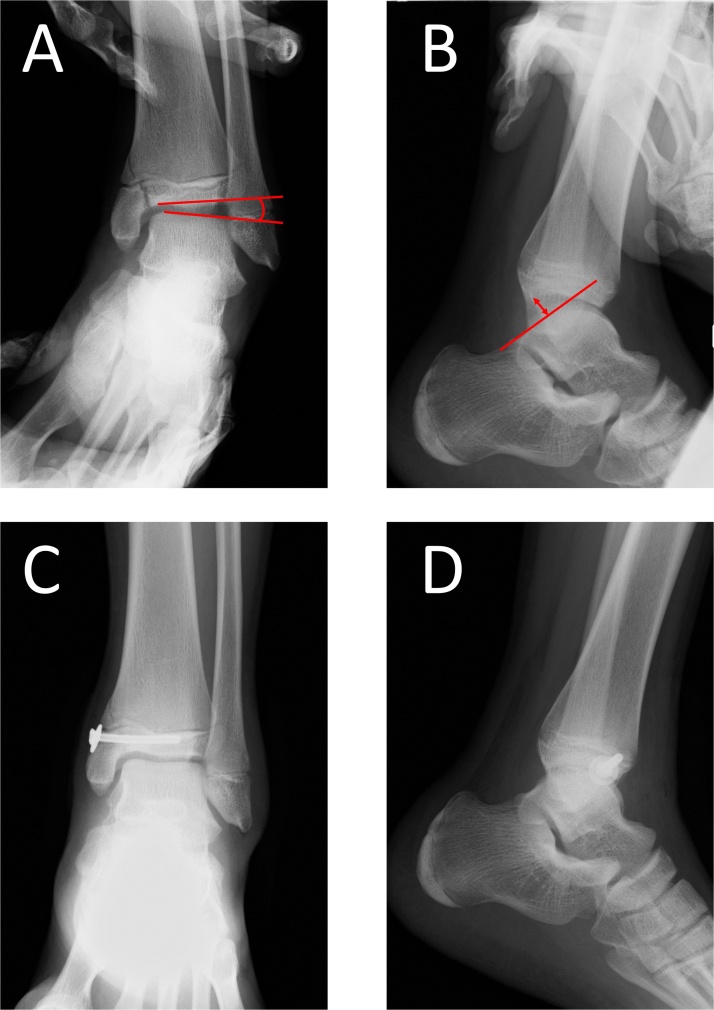
Intraoperative varus and anterior drawer stress radiographs showing that the varus talar tilt angle had increased to 8.7° (A) and the anterior talar translation had increased to 8.0 mm (B). Postoperative plain radiographs after internal fixation using a 3.5-mm cannulated screw (C and D).

**Fig. 3:**
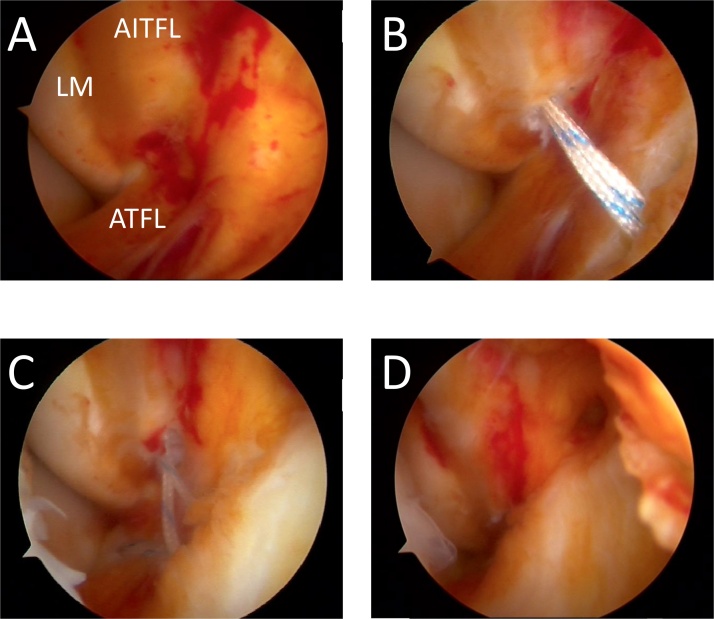
An arthroscopic modified Broström procedure was performed. The anterior talofibular ligament (ATFL) was detached from the footprint at the lateral malleolus (LM) which was the inferior border of the anteroinferior tibiofibular ligament (AITFL) (A). A suture anchor was inserted into the footprint (B). Suture relay was done using the modified lasso-loop stitch technique [8] (C); then ATFL was reattached to the footprint (D).

Surgery was performed under general anethesia by K.Y. who is a specialist of foot and ankle surgery. Varus and anterior drawer stress radiographs showed that the varus talar tilt angle had increased to 8.7° and the anterior talar translation had increased to 8.0 mm (Fig. 2A and B). After oblique drilling of the bone sclerosis at the fracture site with a 1.2-mm Kirschner wire, internal fixation was accomplished using a 3.5-mm cannulated screw (Fig. 2C and D). Thereafter, the arthroscopic modified Broström procedure was performed. First, the midline medial portal just lateral to the tibialis anterior tendon was used for viewing portal. The accessory anterolateral working portal was made by transillumination of the skin in order to protect the superficial peroneal nerve. Subsequently, the suture anchor was introduced into the inferior edge of the anteroinferior tibiofibular ligament, which is the footprint of the lateral talofibular ligament. The lateral talofibular ligament was reattached to the footprint using the modified lasso loop stitch technique [8] (Fig. 3).

**Fig. 4:**
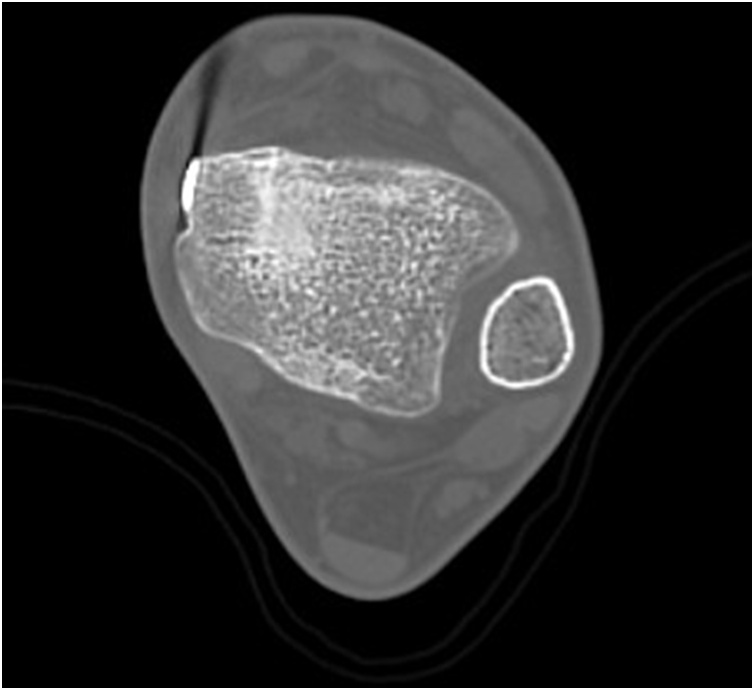
Three months postoperatively, computed tomography showed complete bone union.

**Fig. 5:**
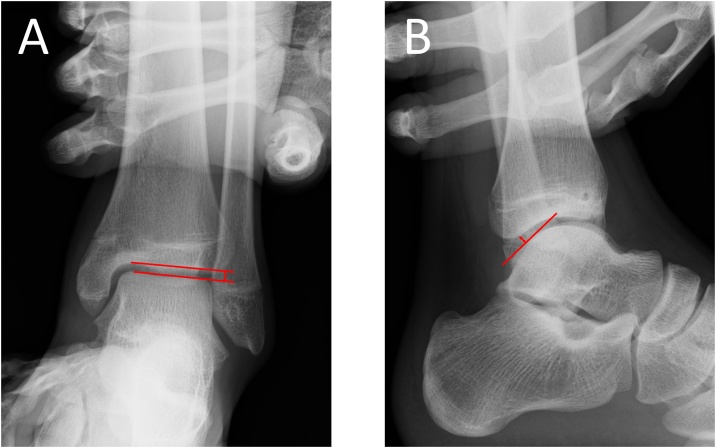
Varus and anterior drawer stress radiographs at 2 years postoperatively improved to 0.8° for varus talar tilt (A) angle and 3.0 mm for anterior talar translation (B).

Postoperatively, weightbearing was disallowed for 4 weeks, with a cast for the initial 2 weeks. The patient gradually progressed to full weightbearing within 4 weeks. Three months postoperatively, CT showed complete bone union (Fig. 4), and the patient returned to football. The Self-Administered Foot Evaluation Questionnaire [10,11] at 2 years postoperatively markedly improved in all parameters (Table 1). Varus and anterior drawer stress radiographs at 2 year postoperatively had improved to 0.8° for the varus talar tilt angle and 3.0 mm for the anterior talar translation (Fig. 5).

**Table 1:** Self-Administered Foot Evaluation Questionnaire before surgery and at two years after surgery.

	Preoperative	Two years after surgery
Pain	33.9	93.3
Physical functioning	6.8	95.5
Social functioning	12.5	100
Shoe related	33.3	100
General health	70	100
Sports	0	91.7

## 3 Discussion

Diagnosis of medial malleolar stress fractures is difficult because of their vague symptoms and unclear onset. Three basic criteria for the evaluation of medial malleolar stress fractures were established by Retting et al. [12]: tenderness over the medial malleolus and joint effusion; pain during activities before an acute episode; and a vertical line from the tibial plafond. In addition to these criteria, early MRI studies are recommended when medial ankle pain has been present for >1 month [13]. The findings from early radiographs are often normal and radiographic findings might not be present for up to several months after the initial injury. In contrast, MRI has been shown to have excellent sensitivity for diagnosing stress fractures [14]. In this case, early MRI might have enabled early diagnosis and treatment and could have prevented nonunion.

Stress fractures develop when normal bone is subjected to repeated stress. It is important to identify the causes of stress fractures to avoid the recurrence of fractures. Soni et al. [15] reported the occurrence of bilateral medial malleolar stress fractures due to osteoarthritis of the knee. In their report, varus malalignment of the knee secondary to medial knee osteoarthritis increased the stress to the medial malleolus of the tibia, and led to bilateral medial malleolar stress fractures. Lee et al. [16] reported medial malleolar stress fracture secondary to CLAI, which was similar to the present case. They performed internal fixation and the arthroscopic Broström procedure, and the patient returned to full participation in baseball 3 months postoperatively. Jowett et al. [5] hypothesized that medial malleolar stress fractures resulted from chronic anteromedial ankle impingement. They reported a small case series of athletes with this injury and concurrent evidence of anteromedial bony spurring of the distal tibia. In their cases, one patient underwent the Broströme procedure for CLAI. Their reported medial malleolar stress fractures might be associated with CLAI, because anterior ankle impingement often occurs secondary to CLAI [17].

Although only one case report [16] has described the relationships between medial malleolar stress fracture and CLAI, we could hypothesized that CLAI could result in medial malleolar stress fracture. The vertical fracture line in the medial malleolus is similar to that seen in Lauge-Hansen supination-adduction injury [18]. Three-dimensional kinematic analysis during gait demonstrated greater rearfoot inversion in patients with CLAI compared to normal groups [19]. Abnormal rearfoot inversion movement is similar to supination-adduction injury, which can cause repetitive stress to the medial malleolus and a vertical fracture. In the present case, we performed arthroscopic Broström procedure for CLAI in addition to internal fixation because we considered that treatment for the cause of stress fracture could prevent fracture recurrence. However, further study is needed to clarify that treatment for CLAI could prevent medial malleolar stress fracture recurrence and lead to better clinical outcome.

## 4 Conclusion

Stress fractures of the medial malleolus are rare, and they can be misdiagnosed or overlooked. Because most of these fractures occur in athletes, early diagnosis with MRI and proper treatment are important for early sports recovery. Because CLAI could potentially induce medial malleolar stress fracture, internal fixation with stabilization of CLAI could be useful for prompt return to sports without fracture recurrence.

## Declaration of Competing Interest

The authors report no declarations of interest.

## Funding

This research did not receive any specific grant from funding agencies in the public, commercial, or not-for-profit sectors.

## Ethical approval

This study was approved by our institutional review board (IRB number 130).

## Consent

We promise that written informed consent was obtained from the patient for publication of this case report and accompanying images. Patient’s name, initial, or hospital numbers were not used in manuscript.

## Authors contribution

Kensei Yoshimoto and Masahiko Noguchi contributed to the conception and design of the case report, decision regarding the treatment protocol, acquisition of data, and manuscript preparation. Hideyuki Maruki and Mina Ishibasi contributed to the decision regarding the treatment protocol and participated in the surgery. Ken Okazaki made valuable suggestions regarding the design and conception of the case report. All authors critically reviewed the manuscript, approved the final version of the manuscript, and have agreed to be accountable for all aspects of the work in ensuring that questions related to the accuracy or integrity of any part of the work are appropriately investigated and resolved.

## Registration of research studies

Not applicable.

## Guarantor

There are no guarantor except for listed authors.

## Provenance and peer review

Not commissioned, externally peer-reviewed.
